# Insulin Resistance: Any Role in the Changing Epidemiology of Thyroid Cancer?

**DOI:** 10.3389/fendo.2017.00314

**Published:** 2017-11-14

**Authors:** Roberta Malaguarnera, Veronica Vella, Maria Luisa Nicolosi, Antonino Belfiore

**Affiliations:** ^1^Endocrinology, Department of Health Sciences, University Magna Graecia of Catanzaro, Catanzaro, Italy; ^2^School of Human and Social Sciences, “Kore” University of Enna, Enna, Italy

**Keywords:** insulin resistance, insulin, thyroid cancer, obesity, type 2 diabetes, insulin growth factor, metformin, insulin sensitizers

## Abstract

In the past few decades, the incidence of thyroid cancer (TC), namely of its papillary hystotype (PTC), has shown a steady increase worldwide, which has been attributed at least in part to the increasing diagnosis of early stage tumors. However, some evidence suggests that environmental and lifestyle factors can also play a role. Among the potential risk factors involved in the changing epidemiology of TC, particular attention has been drawn to insulin-resistance and related metabolic disorders, such as obesity, type 2 diabetes, and metabolic syndrome, which have been also rapidly increasing worldwide due to widespread dietary and lifestyle changes. In accordance with this possibility, various epidemiological studies have indeed gathered substantial evidence that insulin resistance-related metabolic disorders might be associated with an increased TC risk either through hyperinsulinemia or by affecting other TC risk factors including iodine deficiency, elevated thyroid stimulating hormone, estrogen-dependent signaling, chronic autoimmune thyroiditis, and others. This review summarizes the current literature evaluating the relationship between metabolic disorders characterized by insulin resistance and the risk for TC as well as the possible underlying mechanisms. The potential implications of such association in TC prevention and therapy are discussed.

## Introduction

Thyroid cancer (TC) is a relatively rare cancer but represents one of the most common malignancies originating from the endocrine organs (1). It is more frequent in women than in menand is now the third most common cancer in women under the age of 45 in highly developed countries (2). Among various histotypes, differentiated thyroid carcinomas (DTCs) are the most frequent, accounting for approximately 85% of all TCs (3). Increasing incidence of DTCs has been observed worldwide (4–6) in both men and women, although the cancer-specific mortality remains stable (7, 8). TC incidence has increased about twofold in some European countries (7, 9) and up to threefold in North America in the past decades (7, 10). Some studies put emphasis on the supposed “overdiagnosis” of TC consequent to the widespread use of ultrasonography and fine needle biopsy, and point out to the increasing diagnosis of papillary thyroid microcarcinomas (tumors with a diameter of 1 cm or less) (4, 11, 12). However, other studies (5, 6) have reported an increased incidence of TC of all sizes, suggesting that “overdiagnosis” cannot explain all the findings and that TC incidence is truly increasing. A promising hypothesis is that some rising risk factors might favor the molecular alterations typical of papillary TCs (PTCs), thus increasing its incidence.

The known non-modifiable risk factors for TC are age, sex, ethnicity, and genetic predisposition for TC (13–15). However, epidemiological studies suggest that TC incidence is largely dependent on modifiable risk factors, such as environmental carcinogens, diet habits, and lifestyle (16). Environmental pollutants, such as heavy metals, compounds used by industries, non-anthropogenic carcinogens of volcanic origin (17–19), as well as dietary factors (20), and obesity (21) are some of the putative risk factors suspected to play a role in the changing epidemiology of TC. Interestingly, this increasing incidence involves virtually only the papillary histotype, suggesting that some carcinogens may favor specific molecular abnormalities related to this histotype (5, 22).

## Insulin Resistance, Hyperinsulinemia, and Epidemiologic and Clinical Aspects of TC: A Possible Link?

### Evidence of a Positive Association between TC and Insulin Resistance

Obesity is the most common metabolic disorder associated with insulin resistance and compensative hyperinsulinemia. Obesity has more than doubled its prevalence in the past 30 years reaching a prevalence of 40% in the United States and 30% in Europe. The association of obesity with several cancer histotypes is now well established and has become an area of raising concern in oncology (23). In particular, cancers associated with obesity also pose a therapeutical challenge because they tend to be resistant to conventional as well as to target treatments, to metastasize earlier and to have a worse prognosis (24–26). Approximately 14% of cancer-related deaths in men and 20% in women are partially attributed to obesity.

During the past two decades, several epidemiological studies, although not specifically designed for TC, have consistently suggested that a positive association exists between obesity and TC risk (Table 1). A pooled analysis of 12 case–control studies provided early evidence that body mass index (BMI) and weight at diagnosis were directly related to a higher risk for TC in women [odds ratio (OR) = 1.2 for the highest tertile], but not in men. This association was observed for both PTCs and follicular TCs and in all age groups, although there was a significant heterogeneity between the studies analyzed (27). From 2001 to 2010 several single cohort, case–control, prospective cohort, and cross-sectional studies have confirmed the association between overweigh/obesity and TC risk, although the results are rather inconsistent in men, likely for the smaller number of cancer cases in men and the suboptimal adjustment for potential concomitant risk factors (28–36). However, a meta-analysis based on prospective observational studies, found a positive role of obesity as risk factor for TC in both sexes [relative risk (RR) of 1.33 and 1.14, respectively, for women and men, for each 5-unit increase in BMI] (32). In a prospective study based on self-reported medical history, anthropometric and behavioral factors in 90,713 US radiologic technologists followed for 23 years, an elevated risk for TC was observed for women with a RR of 1.74 (95% CI: 1.03–2.94, *P*-trend: 0.04) for BMI ≥ 35.0 vs. 18.5–24.9 kg/m^2^. A similar association was found for men (37).

**Table 1:** Studies regarding a possible association between TC risk and insulin-resistance and related disorders.

Conditions	Reference
Insulin-resistance	Rezzónico et al. (38)
	Bae et al. (39)

Obesity	Ron et al. (40)
	Dal Maso et al. (27)
	Samanic et al. (28)
	Oh et al. (41)
	Engeland et al. (29)
	Renehan et al. (32)
	Brindel et al. (33)
	Clero et al. (35)
	Leitzmann et al. (36)
	Kitahara et al. (21)
	Almquist et al. (42)
	Rinaldi et al. (43)
	Kim et al. (44)
	Oberman et al. (45)

T2DM	Wideroff et al. (46)
	Meinhold et al. (37)
	Chodick et al. (47)
	Aschebrook-Kilfoy et al. (48)
	Duran et al. (49)
	Lai et al. (50)
	Tseng (51)
	Paulus et al. (52)
	Yeo et al. (53)
	Oberman et al. (45)

In 2011, a pooled analysis of five prospective studies including a large number of incident TC in men, and taking into account several potential risk factors, found that the risk of TC was greater with increased BMI [per 5 kg/m^2^: hazard risk (HR) in women 1.16 and 1.21 in men]. When considering women and men together, the HR was 1.2 and 1.53, respectively, in overweight and obese subjects. No differences were found among the TC histotypes. This pooled analysis provided the first strong support to the concept that obesity is an independent risk factor for TC in both women and men (21). However, these studies have limitations, as they lack data on fat distribution, amount of lean versus fat mass, fat mass and/or insulin resistance-related biomarkers, and thyroid function parameters. These technical issues and the low HR values often reported impose caution in interpreting the biological significance of these results.

Three studies conducted in 2012 attempted to provide additional clues regarding TC association with insulin-resistance parameters (43, 54, 55). However, the results were conflicting. Two of these studies (43, 55) found an increased risk of TC in subjects with high waist circumference (>102 cm in men and >88 cm in women), a parameter that correlates with visceral adiposity and is a solid readout of insulin resistance. The HR was 1.79 in men (56) and ranged from 1.42 to 1.54 in women (43), suggesting that central adiposity may impact on TC risk. In contrast, the third study (54), conducted in a cohort of postmenopausal women, failed to find an association between TC risk and various adiposity parameters such as waist circumference, waist-hip-ratio, hip circumference, and BMI (54). In a meta-analysis of seven cohort studies, the combined RR of TC was 1.18 (95% CI: 1.11–1.25) for overweight and obesity combined (57).

Another recent pooled analysis (56) included 22 prospective studies investigating the association between anthropometric factors, such as waist circumference, baseline BMI, and BMI gain and the risk of TC. Data showed that all anthropometric factors analyzed were associated with an increased risk of all histotypes of TC originating from follicular cells: HR for height (per 5 cm) = 1.07; BMI (per 5 kg/m^2^) = 1.06; waist circumference (per 5 cm) = 1.03; young-adult BMI (per 5 kg/m^2^) = 1.13; and adulthood BMI gain (per 5 kg/m^2^) = 1.07. Associations for baseline BMI and waist circumference were mitigated after mutual adjustment (HR for waist = 1.02 and for BMI = 1.01). Furthermore, baseline BMI and BMI gain were strongly associated with anaplastic TC (ATC) and TC mortality.

A strong association between BMI and TC clinical–pathological features has been also confirmed by other studies, which found that, in patients affected by papillary TCs (PTCs), overweight and obesity were positively associated with recurrent or residual post-operative locoregional events (58), extrathyroidal invasion and advanced TNM (TNM Classification of Malignant Tumors) stage (44, 59). Taken together, these results suggest that excess adiposity is associated with increased incidence and mortality for TC of follicular origin. However, at least one study has reported an inverse relation of BMI with stage, tumor invasion and recurrence (60), suggesting that more studies are needed to better evaluate the link between TC prognosis and adiposity.

### Dysregulation of Adipocytokines As a Possible Contributor to Cancer Risk in Obese Patients

**Figure 1:**
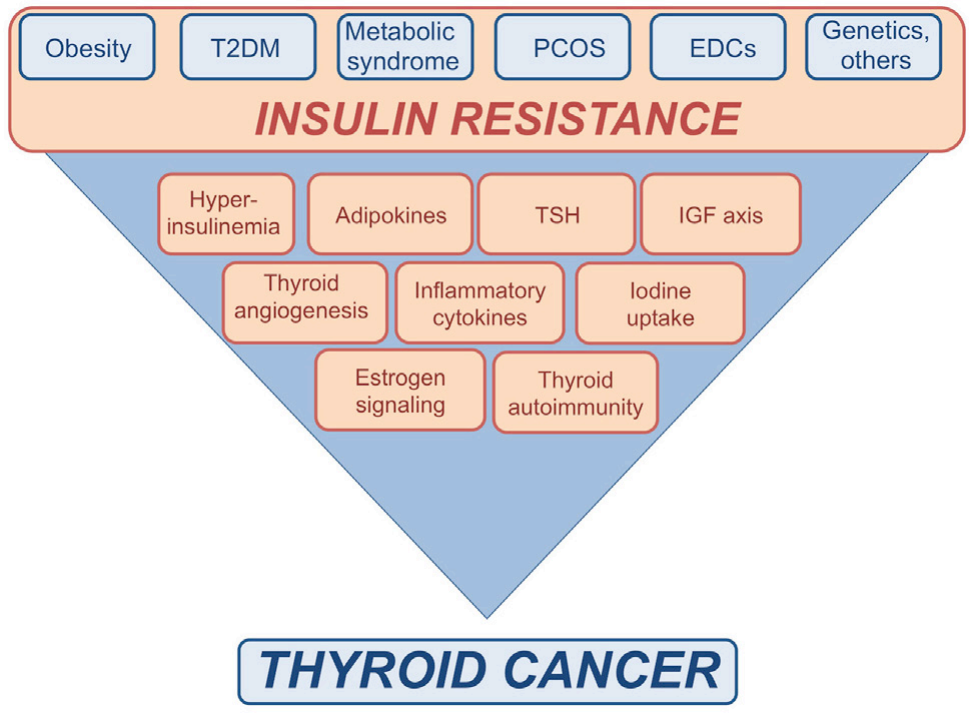
Schematic representation of the possible links between insulin resistance and thyroid cancer (TC). Insulin resistance consequent to metabolic disorders, as well as exposure to endocrine disrupting chemicals (EDCs), genetic factors, and other conditions may affect the risk of TC by inducing or increasing various risk factors.

Obesity is strictly associated not only with insulin resistance and hyperinsulinemia but also with a profound dysregulation of adipocytokines secretion (Figure 1). Indeed, adipose tissue has strongly been established as an endocrine organ for its ability to secrete several polypeptides, known as adipokines, which contribute to the pathogenesis of insulin-resistance and related metabolic alterations in obese patients. Two most known adipokines are leptin and adiponectin. Both of them have been studied as potential contributors to the pathophysiology of cancer associated with insulin resistance, beyond their well-known role in energy balance (61). Leptin is generally up-regulated with increasing fat mass and acts as an antiappetite regulator, mainly through specific membrane receptors, the obesity receptors (Ob-Rs). Aberrant expression of leptin and/or its receptor have been found in a variety of malignancies including TC (62, 63). *In vitro* studies have shown that leptin modulates growth, proliferation and invasion of TC cell lines *via* activation of various prosurvival signaling pathways such as Janus kinase/signal transducers of activated transcription (JAK/STAT), phosphoinositide-3-kinase (PI3K)/protein kinase B/Akt (PKB/Akt), and/or mitogen-activated protein kinase (MAPK) (62, 63). However, the results have been sometimes contradictory, likely because of dependence on the cell type and cell context.

Adiponectin is the most abundant adipokine negatively correlated with body fat, BMI, insulin-resistance, and inflammation states (61). Adiponectin binds two receptors isoforms (AdipoR1, AdipoR2) and acts as an insulin-sensitizer, anti-inflammatory and anti-tumor agent, the latter by inhibiting cell proliferation and angiogenesis and increasing apoptosis *via* the involvement of mammalian target of rapamycin (mTOR)/5′ adenosine monophosphate-activated protein kinase (AMPK), MAPK, JAK/STAT, and PI3K/PKB/Akt pathways (61, 64). So far, few studies have investigated the association between adiponectin and TC. One of these studies has shown that TC specimens and cell lines express both AdipoR1 and AdipoR2. However, in the TC cell lines evaluated, recombinant adiponectin did not exert significant biological effects (65).

For both adiponectin and leptin only a limited number of *in vivo* studies have been performed. Serum leptin levels in papillary thyroid tumor patients were found to be significantly higher than in control subjects, and Ob-Rs expression in TC tissues was significantly associated with more aggressive tumor phenotype (66, 67). However, these studies did not find significant differences in BMI between cancer patients and control subjects (66–72).

Adiponectin levels have been found to be lower in TC patients than in controls (17.00 ± 6.32 vs. 19.26 ± 6.28 µg/ml) (65). Besides, individuals in the highest tertile of adiponectin levels showed a lower risk for TC (OR = 0.29; 95% CI: 0.14−0.55) (65). Conversely, in a prospective cohort study of patients with end-stage renal disease, low adiponectin levels were an independent predictor of developing cancer, with the TC being the second more common malignancy (71). Yet, in a single-cohort study of patients affected by PTC, tumor expression of adiponectin receptors (both AdipoR1 and AdipoR2) was positively correlated with the tumor aggressiveness.

In summary, several studies suggest a significant association between obesity, visceral adiposity and altered adipocytokine profile with TC risk and aggressiveness. However, at least some of these studies have significant limitations with regard to study design, including lack of adjustment for potential confounders, and/or limited statistic power. Therefore, more studies are needed to confirm these conclusions. As a practical implication of these data, one study found that in obese patients with established risk factors [family history, radiation exposure, Hashimoto’s thyroiditis (HT), elevated thyroid stimulating hormone (TSH)], ultrasound screening for TC is cost-effective (73).

## The Interplay of Insulin Resistance with Other Putative Risk Factors for TC

### Insulin Resistance and TSH

In follicular well-differentiated thyroid cells, signaling mediated by pituitary TSH represents the major pathway, which primes thyroid cells to undergo cell cycle progression, DNA synthesis, and cell proliferation (74). The key role of TSH signaling in thyroid carcinogenesis is supported by large epidemiological studies showing a strong association between serum TSH levels and TC development and progression (75–77) (Figure 1). However, even in differentiated hystotypes, suppression of TSH is not enough to avoid or block local invasion and distant metastases. This observation suggests that the mitogenic effect of TSH on human thyrocytes is modulated by other factors including insulin, insulin growth factor-1 (IGF-1), insulin growth factor-2 (IGF-2), and epidermal growth factor (EGF) (74, 78–83).

Classically, TSH induced growth in thyrocytes occurs mainly through the TSHR-dependent increase in cyclic adenosine monophosphate (cAMP), which in turn activates protein kinase A (PKA)-dependent and -independent pathways including: cAMP/PKA/cAMP response element-binding protein (CREB), cAMP/PKA/exchange factor directly activated by cAMP 1/Ras-related protein 1(Rap1)/extracellular signal-regulated kinases (ERKs)/ETS transcription factor, protein kinase C (PKC)/nuclear factor kappa-light-chain-enhancer of activated B cells, nuclear factor κB, PKC/proto-oncogene c-Raf (c-Raf)/ERK/MAPK-activated protein kinase-1 (p90rsk), and rat sarcoma virus protein (Ras)/c-Raf/ERK cascades (84–87).

Moreover, full activation of mitogenesis results from the crosstalk between TSH downstream pathways with other signaling networks, such as PI3K/Akt/mTOR, serine/threonine-protein kinase B-Raf, (B-Raf)/MAPK, proto-oncogene protein Wnt-1, (Wnt)/β-catenin, activated by several tyrosine kinase receptors (RTKs) (85, 88). Studies carried out in normal and tumor thyrocytes have especially highlighted the importance of the functional crosstalk between TSH-cAMP and insulin/IGF axis, which occurs at multiple levels (74, 89).

Notably, the IGF axis plays an important role in regulating normal growth and development in the thyroid (90, 91), partially by modulating the expression of thyroid transcription factor 2, which mediates the transcription of thyroid specific genes such as thyroglobulin (Tg) and thyroperoxidase (TPO) (74, 92–95). As mentioned above, the crosstalk between TSH and insulin/IGF axis appears also to play a role in thyroid tumorigenesis. Indeed, in TC cells and tissue specimens, both IGF-1 receptor (IGF-1R) and insulin receptor (IR) are often overexpressed, representing an early event in thyroid carcinogenesis (96, 97). IR expression is also stimulated by TSH, *via* cAMP (98). IR, exists in two isoforms (IR-A and IR-B), and in cancer is predominantly expressed as the “promitogenic” isoform A (IR-A), which binds with high affinity not only insulin but also IGF-2 (97, 99–101). In TC, the activation of the autocrine IGF-2/IR-A loop was found to correlate with cellular dedifferentiation and tumor progression and aggressiveness. Indeed, the relative abundance of IR-A is approximately 40% in normal thyrocytes and increases to over 70% in TC cells with undifferentiated or stem-like phenotype (90, 97) that also produce IGF-2 (97). Interestingly, IGF-1R expression is also high in differentiated cancers but decreases somehow with cancer dedifferentiation (102–105). In agreement with these data, phosphorylated IGF-1Rs are highly expressed in the majority of TCs but tend to be low in aggressive tumors (106). Interestingly, IGF-1R expression in PTC appears to be higher in patients with type 2 diabetes mellitus (T2DM) than in non-diabetic patients (107). Taken together, these data suggest that both IGF-1R and IR-A play a role in TC. However, the IGF-2/IR-A loop appears to be more important than the IGF-1/IGF-1R loop in thyroid cells with dedifferentiated and stem-like phenotype (108) involved in tumor progression and metastasis (90).

In insulin resistant subjects, the crosstalk between TSH and IGFs axis appears to be enhanced (Figure 1). In fact, obese subjects often show TSH levels at the upper limit of the normal range or slightly increased (109) that seem in relation with the degree of obesity and to the levels of cytokines and other inflammatory markers produced by adipose tissue, including leptin (109–117). Although the actual cause for the hyperthyrotropinemia in obese individuals is still unknown, several mechanisms have been proposed, including increased production of pro-TRH by leptin (118), impaired feedback due to decreased T3 receptors in the hypothalamus (119), changes in peripheral deiodination process of thyroid hormones (119–121), the adaptive response to increased energy expenditure, and chronic low-grade inflammation associated with insulin resistance (122).

A relationship between TSH and insulin-resistance has been also reported in women with polycystic ovary syndrome (PCOS), where mild TSH elevation may be positively related to their metabolic phenotype (123–127). Yet, TSH was also positively correlated with HOMA-IR and BMI in type 2 diabetic patients and in patients with metabolic syndrome (128–134). Although these results have been sometimes controversial, overall they support the positive correlation between insulin resistance and increased TSH serum levels (109) (Table 2).

**Table 2:** Studies showing a possible interplay between insulin resistance related disorders and some TC risk factors.

Disorders/TC risk factors	Obesity	T2DM	Metabolic syndrome	PCOS
Increased TSH levels	Iacobellis et al. (111), Radetti et al. (112) Reinehr and Andler (114), Reinehr et al. (115) Michalaki et al. (116), Sari et al. (113), Javanthi et al. (117)	Javanthi et al. (117), Javanthi et al. (128), Wolide et al. (129), Taneichi et al. (130)	Roos et al. (132), Lai et al. (131), Siemimska et al. (133), Mehran et al. (134)	Mueller et al. (123), Benetti-Pinto et al. (124), Benetti-Pinto et al. (135), Trummer et al. (125), Yin et al. (126), Sinha et al. (127)

Iodine deficiency	Lecube et al. (136), Soriguer et al. (137), Eray et al. (138)	Al-Attas et al. (139)		

EDCs	Rundle et al. (140), Fierens et al. (141), Lim et al. (142), Lee et al. (143), Wolf et al. (144)	Fierens et al. (141), Lim et al. (142), Henriksen et al. (145), Lee et al. (143), Lee et al. (146), Wolf et al. (144), Kramer et al. (147), Weinmayr et al. (148), Jerrett et al. (149), Coogan et al. (150), Lang et al. (151)	Lim et al. (142), Lee et al. (152), Lee et al. (153), Wolf et al. (144)	Kandaraki et al. (154), Akin et al. (155), Tarantino et al. (156), Rajkhowa et al. (157), Takeuchi and Tsutsumi (158), Takeuchi et al. (159), Miao et al. (160)

AIT	Michalaki et al. (116), Rotondi et al. (161)	Akbar et al. (162), Yasmin et al. (163), Toulis et al. (164), Sarfo-Kantanka et al. (165)		Al Saab and Haddad (166), Janseen et al. (167), Menon and Ramachandran (168), Garelli et al. (169), Kachuei et al. (170), Sinha et al. (127), Novais Jde et al. (171), Arduc et al. (172), Ganie et al. (173)

Thyroid angiogenesis		Wang et al. (174)		

Hyperinsulinemia itself, a major characteristic of insulin-resistant patients, is considered a determinant of cancer initiation/progression in diabetic/obese patients (175, 176). In the animal model, several studies carried out in hyperinsulinemic male mice overexpressing a dominant-negative, kinase-dead IGF-1R in muscle (MKR mice), have supported the important role of chronic hyperinsulinemia in cancer progression (177, 178). Notably, hyperinsulinemia may increase the growth of orthotopic mammary tumors through direct stimulation of the IR and without the involvement of the IGF-1R (179). However, no such studies have specifically addressed the role of hyperinsulinemia in TC.

Hyperinsulinemia may increase the bioavailability of IGF-1 and IGF-2 by inhibiting the synthesis of IGF-binding protein 1 and 2 and by intensifying IGF-1 hepatic production. The increased bioavailability of IGFs may contribute to tumor progression through the stimulation of IGF-1R, IR/IGF-1R hybrids, and IR-A itself (101). Yet, hyperinsulinemia, by directly activating IR-A, may favors its “non metabolic” functions and the induction of the pro-mitogenic MAPK/mTOR branch. Non-classical molecular partners, such as discoidin domain receptor 1 and G protein-coupled estrogen receptor, can be further recruited by the IGF system activated receptors, thereby favoring cancer cell proliferation and migration (180–183).

Taken together, these studies suggest that, in insulin-resistant patients, the concomitance of increased TSH levels, deregulation of the IGF axis, and hyperinsulinemia, may represent significant risk factors for TC.

### Insulin Resistance and Thyroid Angiogenesis

Recently, a study has suggested that insulin-resistance may affect the growth and progression of thyroid nodules by increasing angiogenesis and intranodular vascularization (Figure 1) (174). Indeed, it was found that insulin-resistance and high HbA1c are positively associated with a predominant intranodular flow, and with velocity, pressure and density of intranodular blood vessels, especially in nodules of large size (174) (Table 2). The molecular mechanisms responsible for these findings warrant further investigation. However, it is possible to speculate that insulin may stimulate vascular endothelial growth factor (VEGF) expression and promote proliferation of vascular endothelial cells in thyroid nodules and tumors, as shown in other contexts (184). In line with these findings, in breast cancer patients, both hyperinsulinemia and hyperglycemia may stimulate the secretion of pro-inflammatory factors, such as tumor necrosis factor-α, tumor growth factor-α, tumor growth factor-β, interleukin-8, fibroblast growth factor-2, and VEGF-α, thus contributing to tumor neoangiogenesis (185).

### Insulin Resistance and Iodine Deficiency

Iodine is essential for the synthesis and regulation of thyroid hormones. The relationship between iodine intake and TC is complex, as both iodine deficiency and iodine excess have been related to TC development (186, 187). Long-term iodine deficiency has especially been associated with follicular and anaplastic histotypes but also with the papillary histotype (188–190). In animal models, iodine deficiency acts as a weak initiator but a strong promoter of TC, mainly of the follicular type (187). The mechanisms linking the association between iodine deficiency and TC are multiple. Severe iodine deficiency may cause increase of TSH levels (191). However, iodine deficiency could *per se* favor angiogenesis in TC tissues by increasing VEGF mRNA expression (192) through the activation of the transcription factor hypoxia inducible factor 1a (192). In TC, iodine deficiency may also activate additional signals such as the mTOR/p70S6K pathway (193). Low iodine levels may also promote TC development favoring H_2_O_2_-mediated radical reactive oxygen species (ROS) generation, which could result in DNA damage and somatic mutations (191).

Iodine deficiency is also linked to insulin resistance. Several lines of evidences have shown that urinary iodine, which is roughly equal to iodine intake, is markedly decreased in T2DM and obese patients as compared to control subjects, and is negatively correlated with glucose, insulin concentrations and HOMA-IR index (136–139, 194, 195) (Table 2). The physiological pathways connecting insulin resistance with iodine status and the molecular mechanisms by which obese individuals show a reduction in urinary iodine levels are still unclear. It has been proposed that inflammatory cytokines secreted by adipose tissue of insulin resistant patients as well as hyperinsulinemia itself may negatively modulate the expression of sodium/iodine symporter (NIS) on the apical surface of enterocytes, thus inducing a decrease in iodine absorption (136). Taken together, these findings suggest a functional association between iodine deficiency and insulin resistance (Figure 1). However, further studies are needed to better clarify the mechanisms underlying this relationship.

### Insulin Resistance and Endocrine Disrupting Chemicals (EDCs)

Various environmental compounds either natural or synthetic, act as EDCs. These substances may affect hormone signaling through different mechanisms. In the thyroid, they may act at different levels: they may interfere with the hypothalamic–pituitary–thyroid axis, induce direct thyroid cell damage, alter peripheral metabolism of thyroid hormones, and/or affect thyrocytes proliferation, increasing the susceptibility to develop DTCs (196). Recently, it has been found that in the volcanic area of Sicily, DTC incidence is abnormally increased possibly through chronic exposure to EDCs of volcanic origin (197), supporting data reported in other volcanic areas (198).

Beyond their intrinsic carcinogenic potential, some EDCs at the concentration found in human plasma, may lead to disturbances in glucose and fat metabolism. Indeed, they alter pancreatic β-cell function in cellular and animal models (199) and inappropriately regulate intracellular lipid homeostasis as well as proliferation and differentiation of adipocytes (200). These observations suggest that some environmental EDCs may represent a risk factor in the etiology of T2DM and other metabolic disorders, particularly in pre-diabetic individuals (199). In support of these evidences, several biological and epidemiological studies have correlated EDCs exposure with obesity, metabolic syndrome, T2DM and other diseases related to insulin-resistance, including cancer (Figure 1) (201, 202) (Table 2). Recently, it has been found that long-term exposure to air pollution is associated with an increase in HOMA index and insulin levels (144, 202). Some EDCs, including certain metals, by disrupting estrogen homeostasis or by mimicking estrogen actions, may lead to a pregnancy-like metabolic state characterized by insulin-resistance and hyperinsulinemia (199). Furthermore, estrogens potentiate insulin proliferative effects (203). Therefore, EDCs may contribute to DTC initiation and progression (204). The observation that DTC is 3-fold more frequent in women than in men (205) support the pivotal role of estrogens in DTC etiopathogenesis. Indeed, 17-β estradiol (E2) is a potent stimulator of benign and malignant thyrocytes, and both estrogen receptor α (ERα) and ERβ are expressed in DTCs (206). Moreover, as it has been seen in other cellular contexts (207–210), it is likely that also in thyrocytes EDCs may induce membrane-initiated rapid signals involving androgen receptors (ARs) and ERs, both of which crosstalk with the IGF axis (211).

In summary, long-term exposure to EDCs is linked to TC development and progression by multiple mechanisms that include direct toxic effects, estrogen-like effects, and worsening of insulin-resistance (Figure 1).

### Insulin Resistance and Chronic Autoimmune Thyroiditis (AIT)

The association between AIT and DTC has long been a topic of controversy. Data available so far are conflicting. The coexistence of these two diseases has been reported by numerous studies ranging from 0.5 to 30% (212). A meta-analysis conducted by Singh et al. (213) demonstrated that the incidence rate of HT, the most common AIT, is 2.8 times higher in patients with PTC than in patients affected by benign thyroid diseases, and that patients with HT were affected by PTC twice as often as expected. However, many of the published studies are retrospective, had used variable histological methodologies and definitions and have been subjected to several selection biases. Furthermore, it should be underlined that population-based fine needle aspiration biopsy studies have not confirmed this relationship between HT and DTC (214).

The presence of chronic inflammation in HT acting as an initiating factor in carcinogenesis could represent a potential mechanism responsible for the link between HT and PTC. Moreover, the increase in TSH levels or in TSH receptor stimulating antibodies (TSAb), the imbalance in the amount of chemokines and cytokines favoring a switch from Th2 to Th1 immune response or the presence of insulin-resistance, may provide additional explanations for this association (215, 216).

A link between insulin-resistance and AIT has been reported by several studies (Table 2; Figure 1). For instance, it has been seen that the prevalence of AIT in insulin-resistant individuals is higher compared to normal control subjects. For instance, in PCOS patients, anti-TPO antibodies are present in 19.6–26.9% when compared with 3.3–8.3% of control patients (127, 166–173), whereas the prevalence of AIT ranges from 10 to 43% in T2DM patients (162–165) and from 12.4% (in children) to 10–16% (in adults) in obese patients (116, 161). Recently, obesity has been proposed to be a risk factor for thyroid autoimmunity (217). Data showed a positive correlation between leptin and AIT (*r* = 0.26; *P* < 0.001), independent of BMI and fat mass, suggesting the hypothesis that high leptin levels may enhance autoimmune thyroid reaction in a context susceptible to Th-1 immune response (217). Despite these intriguing results, some controversy does remain concerning whether and how insulin resistance prompts the development of AIT. So far, available studies have several limitations, such as restricted number of subjects, biases in the selection of patients and controls, differences in study design, and variability in the use of commercially available assays for anti-TPO antibodies.

### Insulin Resistance in the Context of T2DM

Type 2 diabetes mellitus is characterized not only by insulin resistance but also by hyperglycemia with oxidative stress and advanced glycation end products on proteins and macromolecules, as well as by dyslipidemia and chronic low-grade inflammation (218). In some studies T2DM has been associated with increased risk for TC, although the association ratio values were low (75, 76, 219). Indeed, a recent pooled analysis, including five prospective studies from the USA, showed that the hazard ratio for TC was 1.19 (95% CI: 0.84–1.69) in women and 0.96 (95% CI: 0.65–1.42) in men (55) (Table 1).

Many studies have shown an association between glucose metabolism disorders and thyroid morphologic changes in terms of gland echogenicity, goiter and nodules prevalence, and TSH levels (220–223). In a prospective study, T2DM patients showed higher TSH levels than controls (224). T2DM patients had also larger thyroid volumes and an increased prevalence of nodules. Conversely, in a retrospective survey of 1,559 patients with a new diagnosis of TC (from the continuous National Examination Survey, NHANES) an increased prevalence of T2DM was found among patients who were ≤44 years old as compared to control patients (RR 2.32, CI: 1.37–3.66) (52).

Two longitudinal studies showed that a history of T2DM, ascertained by a self-administered questionnaire, is a risk factor for TC (37, 48). In the total cohort, the increase in TC risk was irrelevant, but it was significantly increased in women (HR. 1.46, 95% CI: 1.01–2.10). Case–control and cohort studies conducted in Unites States (37, 45, 48, 225), Canada (226), Europe (46, 227–230), and Asia (231, 232), confirmed an increased TC risk of approximately 20% in diabetic patients, independently of geographic region, study design, and quality analysis. Despite of a high heterogeneity among studies, the observation that the risk is increased among diabetic women, but not among men, has been always confirmed (37, 46, 233). However, the TC risk associated with DM is more evident in the geographic areas of the world with high rates of TC.

A recent study based on a large prospective cohort, the Women’s Health Initiative, reported data at variance with previous results. In this study, 147,934 cancer free women at baseline were followed up for a median time of 15.9 years. No significant association was found between occurrence of TC with diabetes or diabetes treatment (234). Possible explanations for these negative findings include a weak association between TC and diabetes in postmenopausal women, and the lack of information for insulin resistance and hyperinsulinemia. TC risk is also increased in metabolic syndrome characterized by long-standing insulin resistance, confirming the fundamental role of elevated insulin and glucose levels in the pathogenesis of this association (235). Nevertheless, studies on TC risk in T2DM have some limitations because data regarding the metabolic control, the duration of DM, or the presence of chronic complications, have not always been evaluated. Moreover, confounding elements such as treatment, age of patients, comorbidities like obesity, have not always been appropriately taken into account in some studies. For these reasons, results are somehow controversial and should be interpreted with caution.

Several potential mechanisms can be taken into account to explain the association between T2DM and TC including the higher prevalence of abnormal serum TSH levels (236), the effects of elevated insulin and/or glucose levels in affecting cellular energy metabolism [by increasing the intracellular adenosine triphosphate (ATP)/adenosine monophosphate ratio and inactivating AMPK] (237) and immune system (by increasing ROS production and especially nitric oxide) (237, 238) (Figure 1). Moreover, it has been suggested that chronic treatment with some antidiabetic drugs, may favor the association between T2DM and cancer (239).

Clearly, insulin therapy causes chronic peripheral hyperinsulinemia, and several studies have attempted to clarify whether long-term treatment with insulin or insulin analogs may increase the risk of overall cancer mortality and incidence in patients with T2DM (240, 241). Although some studies have suggested that, unlike native insulin, the long-acting insulin analog glargine could be associated with a higher risk for cancer, especially breast cancer (241–246), re-analysis of these data, as well as further studies have found no differences in cancer risk for insulin glargine as compared with native insulin or other insulin analogs (240). Therefore, there is no clear recommendation regarding the use of insulin or insulin analog in relation to cancer risk.

The possible role of insulin secretagogues (sulfonylureas, glinides) has also been studied. Sulfonylureas (SUs) (glibenclamide, glipizide, and glimepiride) are widely used in diabetic patients. Binding to sulfonylurea receptor 1 on pancreatic beta cells, they stimulates insulin release from the intracellular vesicles. Being potent stimulators of insulin secretion, in principle, sulfonylureas might increase cancer risk. However, epidemiological studies have given controversial results sometimes showing increased cancer risk (243, 247–249). Less potent insulin secretagogues, such as glinides do not appear to be associated with cancer risk (250–253). In any case, none of the above-mentioned studies has focused on TC.

Incretin-based therapies include glucagon-like peptide-1 receptor (GLP-1R) agonists and dipeptidyl-peptidase-4 inhibitors, both of which amplify the insulin response to glucose besides having pleiotropic effects. Therapy with GLP-1R agonists has been recently linked to the C-cell hyperplasia and increased medullary, but not follicular, TC in rodents (245, 254). However, this effect has been observed after lifetime exposure to supratherapeutic doses (255). Moreover, as human thyroid tissues express very low levels of GLP-1R this risk seems to be irrelevant. Data from human observational studies and clinical trials have yielded inconclusive results, thus, continuous monitoring of this issue is still required. Patients with T2DM often follow combination therapies with multiple drugs, making these epidemiological studies very difficult. Moreover, many studies have not taken into account the length of treatment, thus introducing time-related bias.

Two main classes of antidiabetic drugs, thiazolidinediones (TZDs) and biguanides act by reducing insulin resistance (insulin sensitizers). Their possible role in TC is discussed below.

Other antidiabetic drugs, such as alpha-glucosidase inhibitors or SGLT-2 inhibitors, do not directly affect insulin levels or insulin resistance. In any case, no data regarding the use of these drugs and the risk of TC are available.

## Possible Implications for TC Prevention and Therapy

Insulin resistance is multifactorial, and genetic factors account for a significant proportion of insulin resistant subjects (256–262). However, physical inactivity and visceral obesity are the most frequent preventable causes of insulin resistance (263–265).

While the underlying biological mechanisms remain to be investigated, insulin resistance seems to be worsened by iodine deficiency in obese and diabetic patients (136–139, 194, 195). Moreover, evidences showing that TSH and estrogens potentiate the growth effects of insulin, lend support to the hypothesis that insulin resistance may significantly affect the risk of TC, especially by interacting with subclinical hypothyroidism, iodine deficiency, and endocrine disruptors with either estrogen-like or antithyroid activity (Figure 1). At least two of these factors, insulin resistance and environmental contamination with endocrine disruptors have been steadily rising in the past decades (204, 266) and it is reasonable to hypothesize that the interplay among these factors may contribute to the worldwide increase of PTCs incidence (16, 17, 20, 21).

Prevention and therapy of visceral obesity and of related disorders, such as T2DM and metabolic syndrome, are the mainstay to limit the spread of insulin resistance in the population. To this aim, and to reduce associated disorders including cancer, several international organizations and scientific societies have issued guidelines that recommend a healthy lifestyle consisting of constant physical activity and a correct diet (267–270). It is worth noting that these lifestyle changes are difficult to attain and maintain for most people and that only a small proportion of obese subjects is able to achieve significant weight loss by these measures. However, relatively small weight losses may bring about significant amelioration of insulin resistance (271, 272). Whether reduction of insulin resistance achieved by lifestyle changes is associated with reduced TC risk is currently unknown.

Exposure to environmental risk factors, such as iodine deficiency and contamination with endocrine disruptors, should be considered as well. Although iodine prophylaxis with iodized salt is now established in most countries, borderline low iodine intake can be still observed, especially in countries where iodine prophylaxis is facultative (187, 273).

Similarly, environmental monitoring for EDCs may reveal geographic areas and places with high EDC contamination (204). Clinical, ultrasound and biochemical screening can now easily diagnose autoimmune thyroid disorders that may also be associated with TC.

The identification of these additional risk factors may allow the recommendation of individualized strategies for TC prevention.

## Insulin Sensitizers: A Possible Role in TC Prevention and Therapy

As stated above, only a minority of patients succeeds to change lifestyle and achieve long-lasting weight loss. Therefore, the use of insulin sensitizers has been proposed to reduce insulin resistance and its complications.

### Metformin

Metformin is by far the insulin sensitizer most studied in cancer prevention and therapy. A primary effect of metformin is the suppression of the hepatic gluconeogenesis and glucose output, and to increase the peripheral glucose uptake, with consequent reduction in insulin resistance and circulating insulin levels (274).

Interestingly, the use of metformin in diabetic patients has been associated with a lower risk for cancer. A recent meta-analysis of 11 independent studies found an overall 30% statistically significant decrease in cancer risk in patients treated with metformin compared with other diabetic treatments with a promising trend for reduction in overall cancer mortality (275). Similar reductions in cancer risk and mortality was also observed in a second meta-analysis that included 32 articles (276). These and other studies have made a good case for metformin repurposing in cancer chemoprevention. However, a note of caution comes from the fact that these studies regard only diabetic patients, are all retrospective, and results need to be adjusted for multiple variables (277).

Moreover, no specific data on TC were available in these studies, although a recent study performed in Taiwan has shown that the risk of TC is also reduced in diabetic patients treated with metformin (278). However, a second case–control study (279) was unable to find a reduced risk for TC in diabetic patients taking metformin.

In some studies, the use of metformin seems to inhibit the growth of thyroid nodules, which is among risk factors for TC (280–282). In one study, the association of metformin to l-thyroxine was shown to inhibit the growth of thyroid nodules more effectively that l-thyroxine alone (280). In another randomized placebo-controlled clinical trial the use of metformin was also associated with the reduction of small solid thyroid nodules (281). Notably, a recent study showed that metformin therapy in subjects with insulin resistance was effective in decreasing thyroid volume and nodule size (282).

Multiple mechanisms may account for the chemopreventive and anticancer effects of metformin in several cancer histotypes and in TC in particular (283, 284). Relevant to thyroid, additional *in vivo* effects of metformin that may be linked with chemoprevention of TC may include lowering of TSH serum levels in diabetic patients (285). In fact, metformin potentiated the effect of l-thyroxine in reducing thyroid nodule volume in patients with multinodular goiter (280). However, there is evidence that the TSH lowering effect of metformin is seen only in patients with treated hypothyroidism, but not in euthyroid patients (286). Further studies are needed to fully clarify the potential of metformin as chemopreventive drug in non-diabetic, insulin resistant, euthyroid patients.

As an additional mechanism, sex steroids and sex steroid-mimicking EDCs may induce membrane-initiated signals involving AR and ERs and activation of the IGF system (211, 287). These effects have been demonstrated in prostate cancer cells, but may also operate in other cells sensitive to sex hormones. Interestingly, these membrane-initiated signals may be inhibited by metformin (288), thus supporting the potential role of metformin in cancer chemoprevention.

Apart for its possible role in cancer prevention metformin may also play a role in cancer treatment.

Anticancer actions of metformin are partially ascribed to its ability to activate the liver kinase B1/AMPK pathway and to suppress ATP production through the inhibition of mitochondrial complex I (289–292). Both actions of metformin contribute to the inhibition of the mTOR pathway, a major regulator of cell growth and proliferation (277). Metformin may also inhibit ERK signaling (293) and Ca(2+)-dependent PKC-alpha/ERK and JNK/activator protein 1 pathways (294, 295). It may also reduce Akt activity through serine phosphorylation of IRS-1 (296).

Other effects include the inhibition of transcriptional activity of CREB transcriptional factor (297) through the induction of the AMPK-dependent phosphorylation of CREB cofactor CRTC2 at Ser171, which causes CRTC2 sequestration in the cytoplasm by binding with 14–3–3 proteins (298, 299). In fact, dephosphorylated CRTC2 translocates into the nucleus, where it contributes to the CREB-dependent transcription by stimulating the formation of the complex CREB—CREB-binding protein—CRTC2 (297).

Notably, in metformin-treated patients, intraparenchimal metformin concentrations are generally significantly higher than metformin concentration in the bloodstream. For example, metformin concentration at the level of the portal vein is much higher than in the peripheral circulation, thus exposing liver to very high metformin levels (300). Many other organs, including salivary glands, stomach, small intestine, kidney as well as other organs/tissues are also able to concentrate metformin in dependence of the expression of organic cation transporters (OCTs), such as OCT1–2–3 and organ-specific metformin metabolism (301). Metformin also concentrates in the mitochondrial matrix by approximately 1,000-folds (302). These high concentrations are believed to play an important anticancer role (302, 303).

In TC cell lines, metformin was able to inhibit proliferation, through the downregulation of cyclin D1 expression and activation of AMPK, which in turn inhibits the p70S6K/pS6 signaling pathway. Moreover, in undifferentiated TC cells cultured as thyrospheres and enriched in stem-like cells, metformin inhibited the effects of insulin on growth and sphere formation, and potentiated the inhibitory effects of doxorubicin and cisplatin (304). The ability of metformin to potentiate the cytotoxic effects of chemotherapeutics *via* AMPK and p53 signaling was confirmed in other studies (305–307). In addition, metformin may inhibit the growth, migration and mesenchymal transition of TC cell lines by inhibiting mTOR (308). Han et al. showed that metformin elicited a dual antiproliferative effect on primary thyroid cultures and TC cells both by reducing circulating insulin and by directly inhibiting cell cycle progression and survival (304). Accordingly, DTCs occurring in metformin-treated diabetic patients were found to be significantly smaller and with increased progression-free survival as compared with the non-metformin groups (309). A higher remission in patients with TCs with cervical lymph node metastasis has also been observed (310).

Taken together, these data suggest that metformin might play a role in prevention and treatment of thyroid nodules and cancer in insulin resistant patients. Several clinical trials are currently under way with the aim to evaluate the efficacy of metformin as an add-on therapy for patients with various cancer histotypes, but none of these is focused on TC (Clinicaltrials.gov).

### PPAR-γ Agonists

Thiazolidinediones, also known as glitazones, bind and activate the nuclear receptors PPAR-γ acting as agonists. They are potent insulin sensitizers used in the treatment of patients with T2DM (311). Although both metformin and TZDs decrease hepatic glucose production (312, 313), only TZDs reduce liver fat content (312, 314) and diminish fasting free fatty acid concentrations (315) thus improving skeletal muscle insulin sensitivity and reducing liver steatosis. However, side effects of TZDs, such as weight gain and fluid retention that can precipitate cardiac failure and bone fractures, have limited their use in clinical practice. Troglitazone and rosiglitazone (RGZ) were withdrawn because of hepatotoxicity (316) and suspected to increase cardiovascular risk (317), respectively. In addition, the benefit–risk ratio of pioglitazone (PIO) has been reassessed recently in light of a putatively increased risk of bladder cancer.

In a population-based study (318), it has been found that RGZ was associated with a 30–50% reduced risk of TC. In dose response analysis, the adjusted hazard ratios (95% confidence intervals) were significant for the third tertile of duration of therapy (≥14 months) and cumulative dose ≥1,800 mg (0.53, CI 0.31–0.89) and for age ≥50 years (0.50, CI 0.29–0.87) (318). However, a successive study using PIO did not show the same protective effect on TC risk, even if some limitations related to the patients classification or the presence of confounding factors cannot be excluded. However, the different results obtained with the two glitazones (RGZ and PIO) suggested that, apart from restoring insulin-sensitivity, the two drugs might have differential mechanisms on cancer (319) and thyroid cells (320).

## Inhibitors of Insulin/IR-A Signaling

Several studies have highlighted the importance of the insulin/IGF-2/IR-A pathway as a potential target in tumors addicted to this signaling (101, 105). However, for several reasons, targeting this pathway in cancer treatment is not simple. In particular, it is now well accepted that IR and its homolog IGF-1R are functionally interconnected by forming hybrid receptors with an important role in cancer (321), and that targeting either IR or IGF-1R alone results in increased activity of the homolog receptor (322) and resistance to treatment. However, various strategies have been developed to target the insulin/IGF-2/IR-A pathway. Whether these approaches may have specific benefits in insulin resistant patients with cancer is unknown.

In order to avoid or minimize the severe derangement of the glucose metabolism associated with inhibition of total IR, future therapies should possibly aim at specific targeting of IR-A. However, specific antibodies or other drugs able to inhibit the IR-A and not the IR-B are not available and difficult to obtain because of the small differences between the two IR isoforms (323).

The identification of mutations in splicing factors in several malignancies (324, 325) has led to the development of drugs able to counteract the effects of these mutated splicing factors (326, 327). However, whether such drugs may inhibit IR-A formation and favor the IR-B isoform in TC is unknown. Another possible approach is to take advantage of the differential regulation of IR isoform protein maturation. Indeed, furin and paired basic amino acid-cleaving enzyme 4 enzymes, seems to be differentially required for IR-A and IR-B maturation (328, 329), and furin can be inhibited by a number of polyphenols (330).

Finally, various miRNAs have been found to be dysregulated in obesity and insulin resistance (331). Studies are needed to assess whether some of these miRNAs may play a role in the altered IR-A expression in cancer and whether they could be useful tools to normalize the IR-A:IR-B ratio.

Currently available small molecule TK inhibitors lack specificity for IR-A, but are able to coinhibit the IR and IGF-1R. The most studied drugs in this category are Linsitinib (OSI-906) and BMS-754807. Preclinical data, showing a significant efficacy of both drugs either alone or in combination therapies (332), have prompted several phase I–III studies—https://clinicaltrials.gov/ct2/results?term=linsitinib&pg=1 and https://clinicaltrials.gov/ct2/results?term=BMS-754807+&Search=Search. However, no definite evidence of efficacy in a clinical setting has been demonstrated so far.

A different approach for malignancies driven by the IGF-2/IR-A loop is to block IGF-2 using specific antibodies or specific ligand traps. A specific trap for IGF-2 can be obtained using a soluble preparation of the high-affinity domain 11 of M6P/IGF-2R (333, 334), while the soluble form of the IGF-1R combined with the Fc portion of IgG1 can provide a trap for both circulating IGF-1 and IGF-2 (335). These therapies have the advantage to block IR-A stimulation by IGF-2 without impairing the metabolic effects of insulin. However, they do not inhibit the effects of high circulating insulin levels in insulin resistant patients. Preclinical studies are encouraging but clinical data are lacking (335). So far, no studies have addressed the question whether inhibition of the insulin/IGF-2/IR-A signaling by these approaches may provide benefits to patients with TC in the context of insulin resistance.

## Summary and Perspectives

Several lines of evidence now support the concept that the activation of the insulin/IR axis plays a role in TC carcinogenesis. In particular, various dysmetabolic conditions characterized by insulin resistance are significantly associated with an increased risk and worse prognosis of TC. Whether and to what extent insulin resistance plays a role in the worldwide, steady increase in PTCs has not been clarified yet. Indeed, several clinical studies performed until now, have reported only a positive association rather than a causative role. Moreover, some of these studies show significant limitations, including lack of adjustment for potential confounders, and/or limited statistic power and/or low OR values. These limitations should be taken into account when considering the physiological/biological significance of these studies. Similarly, more studies are required to elucidate the possible interactions between insulin resistance/hyperinsulinemia and more established TC risk factors, such as radiations, iodine deficiency, endocrine disruptors, and inflammation. Finally, how obesity derived cytokines, and overactivation of the insulin/IGF axis may affect the molecular pathways involved in the pathogenesis of TC should be explored in depth.

However, as for other malignancies associated with insulin resistance, it is to be expected that a correct lifestyle, which includes a healthy diet and physical activity, aimed at preventing obesity and T2DM would exert a beneficial effect also in TC occurrence. For all people living in iodine deficient areas, iodine prophylaxis is mandatory in order to avoid the growth promoting effect of reduced iodine intake on the thyroid gland. It could be hypothesized that additional attention should be lent to people with concomitant insulin resistance in order to avoid the combined effects of hyperinsulinemia and iodine deficiency. In T2DM patients, administration of insulin sensitizers or inhibitors of SGLT2 (336, 337) should be preferred to insulin stimulating drugs and insulin itself. Hopefully, these assumptions will be validated by future studies, although, because of the indolent natural history of most DTCs, such studies may prove to be difficult to perform.

As far as therapy is concerned, surgery, radioactive iodine treatment and TSH suppression by l-thyroxine administration are the cornerstones of DTC treatment. However, no specific therapy exists for poorly differentiated or undifferentiated TCs that have lost the ability to uptake radioiodine. Drugs with multikinase inhibiting activity are increasingly used in these cancers, but so far their effect on cancer mortality is at best uncertain (338). Clearly, new combined therapies are urgently required for these aggressive cancers. Evidences showing that the insulin/IGF axis is frequently activated in these tumors owing to overexpressed IR and IGF-1R and increased local production of IGFs lend support to the possibility that therapies targeting this axis could have a role in these new approaches. The increased awareness that overexpression of IR-A possibly plays a more important role than IGF-1R and may compensate for IGF-1R inhibition, strongly suggests that dual IR and IGF-1R inhibitors should be more efficacious than specific inhibitors of IGF-1R. Moreover, future approaches may explore the efficacy of drugs specifically targeting IR-A or pathways preferentially activated by the IGF-2/IR-A loop. Finally, insulin sensitizers able to reduce peripheral insulin levels could have a role on both prevention and treatment of TC. Certainly, more studies are needed to address the role of insulin and insulin resistance in better and individualized programs of TC prevention and adjuvant therapies. More in general, in spite of several lines of evidence indicating that obesity/insulin resistance-driven mechanisms are associated with cancer development and progression, specific guidelines for cancer prevention and treatment in these patients are lacking and should be considered a desirable aim of precision medicine.

## Author Contributions

VV, RM, MN, and AB: substantial contributions to the conception and design of the article; drafting the work; final approval of the version to be published; and agreement to be accountable for all aspects of the work in ensuring that questions related to the accuracy or integrity of any part of the work are appropriately investigated and resolved. VV, RM, and AB: revising it critically for important intellectual content.

## Conflict of Interest Statement

The authors declare that the research was conducted in the absence of any commercial or financial relationships that could be construed as a potential conflict of interest.

## Abbreviations

AIT, autoimmune thyroiditis; cAMP, cyclic adenosine monophosphate; AMPK, 5′ adenosine monophosphate-activated protein kinase; ATC, anaplastic thyroid cancer; ATP, adenosine triphosphate; BMI, body mass index; B-Raf, serine/threonine-protein kinase B-Raf; CBP, CREB-binding protein; CRCT2, CREB regulated transcription coactivator 2; CREB, cAMP response element-binding protein; DTC, differentiated thyroid cancer; E2, 17-beta estradiol; EDCs, endocrine disrupting chemicals; EGF, epidermal growth factor; ERKs, extracellular signal-regulated kinases; FGF-2, fibroblast growth factor-2; GPER, G protein-coupled estrogen receptor; HR, hazard risk; HT, Hashimoto’s thyroiditis; IGF-1, insulin growth factor 1; IGF-2, insulin growth factor 2; IL-8, interleukin-8; IR, insulin receptor; JAK/STAT, janus kinase/signal transducers of activated transcription;MAPK, mitogen-activated protein kinase; OR, odds ratio; p90rsk, MAPK-activated protein kinase-1; OCT, organic cation transporter; PCOS, polycystic ovary syndrome; PIO, pioglitazone; PI3K, phosphoinositide-3-kinase; PKC, protein kinase C; PTC, papillary thyroid cancer; PKA, protein kinase A; PKB/Akt, protein kinase B/Akt; c-Raf, proto-oncogene; Rap1, Ras-related protein 1; Ras, rat sarcoma virus protein; RGZ, rosiglitazone; ROS, reactive oxygen species; RR, relative risk; RTKs, tyrosine kinase receptors; TC, thyroid cancer; T2DM, type 2 diabetes mellitus; Tg, thyroglobulin; TPO, thyroperoxidase; mTOR, mammalian target of rapamycin; TSH, thyroid stimulating hormone; TZDs, tiazolidinediones; VEGF, vascular endothelial growth factor; Wnt, proto-oncogene protein Wnt.
